# Identifying genotype-phenotype relationships in biomedical text

**DOI:** 10.1186/s13326-017-0163-8

**Published:** 2017-12-06

**Authors:** Maryam Khordad, Robert E. Mercer

**Affiliations:** Department of Computer Science, University of Western Ontario, 1151 Richmond Street, London, N6A 5B7 Canada

**Keywords:** Genotypes, Phenotypes, Genotype-phenotype relationship, Semi-automatic corpus annotation, Self-training, Computational linguistics

## Abstract

**Background:**

One important type of information contained in biomedical research literature is the newly discovered relationships between phenotypes and genotypes. Because of the large quantity of literature, a reliable automatic system to identify this information for future curation is essential. Such a system provides important and up to date data for database construction and updating, and even text summarization. In this paper we present a machine learning method to identify these genotype-phenotype relationships. No large human-annotated corpus of genotype-phenotype relationships currently exists. So, a semi-automatic approach has been used to annotate a small labelled training set and a self-training method is proposed to annotate more sentences and enlarge the training set.

**Results:**

The resulting machine-learned model was evaluated using a separate test set annotated by an expert. The results show that using only the small training set in a supervised learning method achieves good results (precision: 76.47, recall: 77.61, F-measure: 77.03) which are improved by applying a self-training method (precision: 77.70, recall: 77.84, F-measure: 77.77).

**Conclusions:**

Relationships between genotypes and phenotypes is biomedical information pivotal to the understanding of a patient’s situation. Our proposed method is the first attempt to make a specialized system to identify genotype-phenotype relationships in biomedical literature. We achieve good results using a small training set. To improve the results other linguistic contexts need to be explored and an appropriately enlarged training set is required.

## Background

Many research experiments are being performed to discover the role of DNA sequence variants in human health and disease and the results of these experiments are published in the biomedical literature. An important category of information contained in this literature is the newly discovered relationships between phenotypes and genotypes. Experts want to know whether a disease is caused by a genotype or whether a certain genotype determines particular human characteristics. This information is very valuable for researchers, clinicians, and patients. There exist some manually curated resources such as OMIM [1] which are repositories for this information, but they do not provide complete coverage of all genotype-phenotype relationships. Because of the large quantity of literature possessing this information, a reliable automatic system to identify these relationships for future curation is desirable. Such a system provides important and up to date data for database and ontology construction and updating, and even for text summarization.

### Related work

#### Identifying relationships between biomedical entities by analyzing only biomedical text

Finding the relationships between entities from information contained in the biomedical literature has been studied extensively and many different methods to accomplish these tasks have been proposed. Generally, current approaches can be divided into three types: Computational linguistics-based (e.g., [2–4]), rule-based (e.g., [5, 6]), and machine learning and statistical methods (e.g., [7, 8]). Furthermore some systems (e.g., [9–11]) have combined these approaches and have proposed hybrid methods.

RelEx [10] makes dependency parse trees from the text and applies a small number of simple rules to these trees to extract protein-protein interactions. Leroy et al. [12] develop a shallow parser to extract relations between entities from abstracts. The type of these entities has not been restricted. They start from a syntactic perspective and extract relations between all noun phrases regardless of their type. SemGen [9] identifies and extracts causal interaction of genes and diseases from MEDLINE citations. Texts are parsed using MetaMap. The semantic type of each noun phrase tagged by MetaMap is the basis of this method. Twenty verbs (and their nominalizations) plus two prepositions, *in* and *for*, are recognized as indicators of a relation between a genetic phenomenon and a disorder. Sekimizu et al. [2] use a shallow parser to find noun phrases in the text. The most frequently seen verbs in the collection of abstracts are believed to express the relations between genes and gene products. Based on these noun phrases and frequently seen verbs, the subject and object of the interaction are recognized.

Coulet et al. [4] propose a method to capture pharmacogenomics (PGx) relationships and build a semantic network based on relations. They use lexicons of PGx key entities (drugs, genes, and phenotypes) from PharmGKB [13] to find sentences mentioning pairs of key entities. Using the Stanford parser [14] these sentences are parsed and their dependency graphs^1^ are produced. According to the dependency graphs and two patterns, the subject, object, and the relationship between them are extracted. This research is probably the closest to the work presented here, the differences being that the method to find relationships is rule-based and the entities of interest include drugs. Direct comparison with our results is difficult because the genotype-phenotype relationships with their associated precision and recall values are not presented separately. Temkin and Gilder [3] use a lexical analyzer and a context free grammar to make an efficient parser to capture interactions between proteins, genes, and small molecules. Yakushiji et al. [15] propose a method based on full parsing with a large-scale, general-purpose grammar.

The BioNLP module [5] is a rule-based module which finds protein names in text and extracts protein-protein interactions using pattern matching. Huang et al. [6] propose a method based on dynamic programming [16] to discover patterns to extract protein interactions. Katrenko and Adriaans [8] propose a representation based on dependency trees which takes into account the syntactic information and allows for using different machine learning methods. Craven [7] describes two learning methods (Naïve Bayes and relational learning) to find the relations between proteins and sub-cellular structures in which they are found. The Naïve Bayes method is based on statistics of the co-occurrence of words. To apply the relational learning algorithm, text is first parsed using a shallow parser. Marcotte et al. [17] describe a Bayesian approach to classify articles based on 80 discriminating words, and to sort them according to their relevance to protein-protein interactions. Bui et al. [11] propose a hybrid method for extracting protein-protein interactions. This method uses a set of rules to filter out some PPI pairs. Then the remaining pairs go through a SVM classifier. Stephens et al. [18], Stapley and Benoit [19], and Jenssen et al. [20] discuss extracting the relation between pairs of proteins using probability scores.

Supervised learning approaches have been used to recognize concepts of prevention, disease, and cure and relations among these concepts. Work using a standardized annotated corpus beginning with Rosario and Hearst [21] and continuing with the work of Frunza and Inkpen [22, 23] and Abacha and Zweigenbaum [24, 25] has seen good performance progress.

An approach to extract binary relationships between food, disease, and gene named entities by Yang et al. [26] has similarities to the work presented here because it is verb-centric.

Most of the biomedical relation extraction systems focus on finding relations between specific types of named entities. Open Information Extraction (OIE) systems aim to extract all the relationships between different types of named entities. TextRunner [27], ReVerb [28], and OLLIE [29] are examples of OIE systems. They first identify phrases containing relations using part-of-speech patterns and syntactic and lexical constraints, and then with some heuristics detect related named entities and relation verbs. PASMED [30] extracts diverse types of binary relations from biomedical literature using deep syntactic patterns. Advanced OIE systems [31, 32] have been proposed to extract nominal and n-ary relations.

Increasing interest in neural network models, such as deep [33], recurrent [34], and convolutional [35] networks, and their applications to Natural Language Processing, such as word embeddings [36] have provided a new set of techniques for relationship identification, some which deal with relationships of a general nature, such as Miwa and Bansal [37], and some which deal with biomedical relationships, such as Jiang et al. [38]. Our method is a more traditional pipeline method—identifying genotypes and phenotypes, and then using surface, syntactic, and dependency features to identify the relationships. So, rather than developing an extensive overview of these neural network models, we instead point the reader to Liu et al.’s excellent summary of these methods [39].

#### Identifying genotype-phenotype relationships using biomedical text and/or other curated resources

The research works mentioned in the previous section have been highlighted because they are concerned with identifying various relations among biomedical entities by analyzing only the natural language context in which mentions of these relations and entities are immersed. There is a vast literature presenting research focussed specifically on the genotype-phenotype relation. Most of this research presents the discovery of novel genotype-phenotype relations based on biomedical evidence and is beyond the intent of this paper and would be out of place to be surveyed here. Incidentally, it is this type of literature that we are interested in mining to extract genotype-phenotype relationships.

While not finding genotype-phenotype relationships, many research works are concerned with a related question: disease-gene relationships. One of the earliest works in this area is that of Doughty et al. [40] which provides an automated method to find cancer- and other disease-related point mutations. The method of Singhal et al. [41] to find disease-gene-variant triplets in the biomedical literature makes strong use of a number of modern natural language tools to analyze the text in which these triplets reside, but this method also uses information mined from all of the PubMed abstracts, the Web, and sequence analysis which requires the use of a manually curated database. Another research work that investigates gene variants and disease relationships is that of Verspoor et al. [42]. Another work that investigates mutation-disease associations is Mahmood et al. [43]. A recent review of algorithms identifying gene-disease associations using techniques based on genome variation, networks, text mining, and crowdsourcing is provided by Opap and Mulder [44].

Other literature reports on techniques to extract genotype-phenotype relationships combining biomedical text mining with a variety of other resources. An example of this type of technique is the pioneering work of Korbel et al. [45]. Being the first to use evidence from biomedical literature, it uses the correlation of gene and phenotype mentions in the text together with comparative genome analysis that depends on a database of orthologous groups of genes to provide gene-phenotype relationship candidates. Novel relationships that were not mined directly from the text are reported. Another type of technique, exemplified by the work of Goh et al. [46] is the integration of curated databases to find genotype-phenotype relationship candidates.

A work by Bokharaeian et al. [47] which is very close to the research presented here uses two types of Support Vector Machines for their learning method and the type of relationship being identified is between single-nucleotide polymorphisms (SNPs) and phenotypes. This work presents three types of association (positive, negative, and neutral) and three levels of confidence (weak, moderate, and strong).

In each of the referred to works, either the presentation of the genotype-phenotype relationship is complicated by being part of a larger relationship, such as in the work of Coulet et al. [4], or the method to suggest the relationship requires information found in manually curated databases, such as the works of Korbel et al. [45], Goh et al. [46], and Singhal et al. [41]. Our work then stands out by being different on each of these fronts: we identify only the genotype-phenotype relationships and we use only the text in the PubMed abstract being analyzed. Also, we are not attempting to find new relationships, rather we are only mining those relationships that occur in the abstract. In addition, we are using a machine learning method that requires human annotated data. We view the method provided in this paper as complementing these other methods in the ways just described.

Briefly then, in this paper we discuss a semi-supervised learning method for identifying genotype-phenotype relationships from biomedical literature. We start with a semi-automatic method for creating a small seed set of labelled data by applying two named entity relationship tools [48] to an unlabelled genotype-phenotype relationship dataset. This initially labelled genotype-phenotype relationship dataset is then manually cleaned. Then using this as a seed in a self-training framework, a machine learned model is trained. It is worth noting that throughout this paper we do not take into account the phenotypes at the subcellular level. The evaluation results are reported using precision, recall and F-measure derived from a human-annotated test set. Precision (or positive predictive value) is the ratio of correct relationships in all relationships found and can be seen as a measure of soundness. Recall (or sensitivity) is the ratio of correct relationships found compared to all correct relationships in the corpus and can be used as a measure of completeness. F-measure combines precision and recall as the harmonic mean of these two numbers.

#### Semi-supervised learning

To train machine learning systems, it is easier and cheaper to obtain unlabelled data than labelled data. Semi-supervised learning is a bootstrapping method which incorporates a large amount of unlabelled data to improve the performance of supervised learning methods which lack sufficient labelled data.

Much of the semi-supervised learning in Computational Linguistics uses the iterative bootstrapping approach, initially proposed by Riloff and Shepherd [49] for building semantic lexicons, which later evolved into the learning of multiple categories [50]. These methods have further transformed to the semi-supervised learning of multiple related categories and relations as a method to enhance the learning process [51].

Instead of using this category of semi-supervised learning, we use a methodology called self-training. Ng and Cardie [52] proposed this type of semi-supervised learning to combat semantic drift [53, 54], a problem with the bootstrapped learning of multiple categories. They used bagging and majority voting in their implementation. A set of classifiers get trained on the labelled data, then they classify the unlabelled data independently. Only those predictions which have the same label by all classifiers are added to the training set and the classifiers are trained again. This process continues until a stop condition is met. For Clark et al. [55] a model is simply retrained at each iteration on its labelled data which is augmented with unlabelled data that is classified with the previous iteration’s model. According to this second method, there is only one classifier which is trained on labelled data. Then the resulting model is used to classify the unlabelled data. The most confident predictions are added to the training set and the classifier is retrained on this new training set. This procedure repeats for several rounds. We adopt this latter methodology in our work.

#### Rule-based and machine learning-based named entity relationship identification tools

Ibn Faiz [48] proposed a general-purpose software tool for mining relationships between named entities designed to be used in both a rule-based and a machine learning-based configuration. This tool was originally tailored to recognize pairs of interacting proteins and has been reconfigured here for the purpose of identifying genotype-phenotype relationships. Ibn Faiz [48] extended the rule-based method of RelEx [10] for identifying protein-protein interactions. In this method the dependency tree of each sentence is traversed according to some rules and various candidate dependency paths are extracted.

This extended method is able to detect the more general types of relationships found between named entities in biomedical text. For example the rule-based system is able to find relationships with the following linguistic patterns, where *PREP* is any preposition, *REL* is any relationship term, and *N* is any noun:

- Entity1 *REL* Entity2; e.g., Genotype *causes* Phenotype
- Relations in which the entities are connected by one or more prepositions:
  - Entity1 *REL (of ∣ by ∣ to ∣ on ∣ for ∣ in ∣ through ∣ with)* Entity2; e.g., Phenotype *is associated with* Genotype
  - *(PREP* ∣ *REL* ∣ *N)* ^+^ (PREP)(REL ∣ PREP ∣ N)* Entity1 *(REL* ∣ *N* ∣ *PREP)* ^+^ Entity2; e.g., *expression of* Phenotype *by* Genotype
  - *REL (of* ∣ *by* ∣ *to* ∣ *on* ∣ *for* ∣ *in* ∣ *through* ∣ *with* ∣ *between)* Entity1 and Entity2, e.g., *correlation between* Genotype and Phenotype.
- Entity1 (/∣∖∣−)Entity2; e.g., Genotype/Phenotype *correlation*.

In addition to the linguistic patterns this method requires a good set of relationship terms. To find protein-protein interaction relationships, a list of interaction terms (a combination of lists from RelEx [10] and Bui et al. [11]) was used by Ibn Faiz to elicit protein-protein interactions. In the work reported below an appropriate set of relationship terms for genotype-phenotype relationships has been developed and used in the rule-based system to recognize this type of relationship.

Ibn Faiz [48] also used his general-purpose tool in a machine learning approach using a maximum entropy classifier and a set of relationship terms appropriate for identifying protein-protein interactions. This approach considers the relationship identification problem as a binary classification task. The Stanford dependency parser produces a dependency tree for each sentence. For each pair of named entities in a sentence, proteins in this case, the dependency path between them, the parse tree of the sentence, and other features are extracted. These features include: dependency features coming from the dependency representation of each sentence, syntactic features, and surface features derived directly from the raw text (the relationship terms and their relative position).

The extracted features along with the existence of a relationship between named entity pairs in a sentence make a feature vector. A machine learning model is trained based on the positive (a relationship exists) and negative (a relationship does not exist) examples. To avoid sparsity and overfitting problems, feature selection is used. Because the maximum entropy classifier and the linguistic dependency and syntactic features are the common foundation for this technique, only an appropriate set of relationship terms need to be provided for genotype-phenotype relationship identification. In the work reported below, the same set of relationship terms as used in the rule-based approach are used in the machine-learning approach.

## Methods

**Fig. 1:**
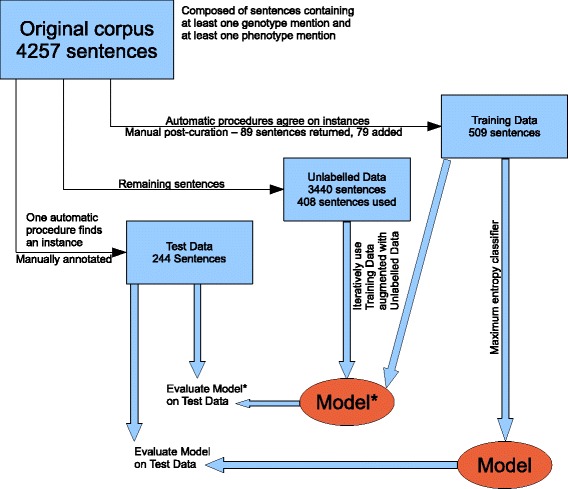
Workflow

A block diagram showing the complete workflow is provided in Fig. 1. Details of this workflow are presented in the following.

### Curating the data

As mentioned before we did not have access to any data prepared specifically for the genotype-phenotype relationship identification task, so our first task was to collect a sufficient number of sentences containing phenotype and genotype names that include both genotype-phenotype relationships and non-relationships. Three sources of data have been used in this project:

- Khordad et al. [56] generated a corpus for the phenotype name recognition task. This corpus is comprised of 2971 sentences from 113 full papers. It is designated as the *MKH* corpus henceforth.
- PubMed was queried for “genotype and phenotype and correlation” and 5160 abstracts were collected.
- Collier et al. [57] generated and made available to us the *Phenominer* corpus which contains 112 PubMed abstracts. Both phenotypes and genotypes are annotated in this corpus, but not their relationships. The annotation was carried out with the same experienced biomedical annotator who accomplished the GENIA corpus [58] tagging. *Phenominer* contains 1976 sentences with 1611 genotypes and 472 phenotype candidates. However, there are two issues with this corpus:
  - The phenotypes at the cellular level are labelled in the *Phenominer* corpus. Our work on genotype-phenotype relationships does not consider this type of phenotype because the linguistic context is different from relationships involving the non-cellular level phenotypes.
    In all of the steps explained below, this type of phenotype is included. We report precision, recall, and F-measure with and without this type of phenotype involved in genotype-phenotype relationships labelled in the test set.
  - Generic expressions (e.g., gene, protein, expression) referring to a genotype or a phenotype earlier in the text are tagged in this corpus as genotypes and phenotypes. For example *locus* is tagged as a genotype in the following sentence: *“Our original association study focused on the role of IBD5 in CD; we next explored the potential contribution of this* ***locus*** *to UC susceptibility in 187 German trios.”*
    The work reported here only considers explicitly named genotypes and phenotypes. Thus, including these examples will have a slightly negative effect on the trained model and any relationships that include entities that are named implicitly will not be identified in the test set, reducing the precision and recall slightly.

Genotype and phenotype names were already annotated in the third resource and phenotypes were already annotated in the first resource. So, we had to annotate genotypes in the first resource and genotypes and phenotypes in the second resource. BANNER [59], a biomedical NER system, has been used to annotate the genotype names and an NER system specialized in phenotype name recognition [56] has been used to annotate the phenotype names. Only sentences with both phenotype and genotype names have been selected from the above resources to comprise our data and the remaining sentences have been ignored. In this way, we have collected 460 sentences from the *MKH* corpus, 3590 sentences from the *PubMed* collection and 207 sentences from *Phenominer*. These 4257 sentences comprise our initial set of sentences. All the sentences are represented by the IOB label model (Inside, Outside, Beginning). The phenotype names and genotype names are tagged by their token offset from the beginning of each sentence because they can occur multiple times in a sentence.

#### Training set

At the beginning of the project we did not have any labelled data. Instead of using annotators knowledgeable in biomedicine to label a sufficiently large corpus of biomedical literature, we decided instead to use the previously described relationship identification tools modified to work with our data and use their agreed upon outputs, cleaned by a non-expert, as our labelled training set. This methodology has allowed us to partially evaluate this method of semi-automatic annotation.

As mentioned previously, the rule-based and machine learning-based systems for identifying biomedical relationships have been appropriately tailored to this task by supplying a set of genotype-phenotype relationship words that are appropriate for identifying this type of biomedical relationship. This set of relationship words includes a list of 20 verbs and two prepositions (*in* and *for*) from Rindflesch et al. [9] which encode a relationship between a genetic phenomenon and a disorder and the PPI relationship terms from Ibn Faiz’s work [48] which we found to apply also to genotype-phenotype relationships.^2^

Our initial corpus is separately processed by the rule-based and the machine learning-based relationship identification tools. Each of these tools find some relationships in the input sentences. After the results are compared, those sentences that contain at least one agreed upon relationship^3^ are initially considered as the training set. From the original corpus, 519 sentences comprised the initial training set as the result of this process. However, as these tools have been developed as general named entity relationship identifiers, we could not be certain that even their similar results produce correctly labelled examples. Therefore, the initial training set was further processed manually. Some interesting issues were observed.

1. Some sentences do not state any relationship between the annotated phenotypes and genotypes. Instead, these sentences only explain the aim of a research project. However, these sentences are labelled as containing a relationship by both tools; e.g., *“The present study was undertaken to investigate whether rare variants of TNFAIP3 and TREX1 are also associated with systemic sclerosis.”*
2. The negative relationships stated with the word “no” are considered positive by both tools; e.g., *“With the genotype/phenotype analysis, no correlation in patients with ulcerative colitis with the MDR1 gene was found.”*
3. Some sentences from the *Phenominer* corpus are substantially different compared to other sentences, because of the two issues we discussed earlier about this corpus. The phenotypes below the cellular level have different relationships with genotypes. For example, they can change genotypes while the supercellular-level phenotypes are affected by genotypes and are not capable of causing any change to them.
4. Some cases have both tools making the same mistakes: suggesting incorrect relationships (i.e., negative instances are suggested as positive instances) or missing relationships (i.e., positive instances are given as negative instances).

After making corrections (see issues 2 and 4) and deleting sentences exhibiting issues 1 and 3, 430 sentences remained in the training set. These corrections and deletions were made by the first author. To increase the training set size, 39 additional sentences have been labelled manually and have been added to the training set. The data set is skewed: there are few negative instances. To address this imbalance, 40 sentences without any relationships have been selected manually and have been added to the training set. As shown in Table 3, the final training set has 509 sentences. There are 576 positive instances and 269 negative instances.

#### Test set

To ensure that the training set and the test set are independent, the test set is chosen from the initial set with the training set sentences removed. To select the sentences to be included in the test set, the results from processing our initial set with the two general purpose relationship identification tools have been used. In some cases both tools identify relationships from the same sentence but the relationships differ. For example in sentence *“Common esr1 gene alleles-4 are unlikely to contribute to obesity-10 in women, whereas a minor importance of esr2-19 on obesity-21 cannot be excluded.”* the machine learning-based tool finds a relationship between *esr2-19* and *obesity-21* but the rule-based tool claims that there is also a relationship between *esr1 gene alleles-4* and *obesity-10*. Since we were confident that this type of sentence would provide a rich set of positive and negative instances, this type of sentence is extracted to make our initial test set of 298 sentences.

In order for the test set to provide a reasonable evaluation of the trained model, the sentences must be correctly labelled. A biochemistry graduate student was hired to annotate the initial test set. Pairs of genotypes and phenotypes are extracted from each sentence and her task was to indicate whether there is any relationship between them.

Issues 1 and 3 discussed in the previous section have been observed by the annotator in some of the sentences. Also, there are some cases where she is not sure if there is a relationship or not. Furthermore, she disagreed with the phenotypes and genotypes annotated in 54 sentences. After deleting these 54 problematic sentences the final test set comprises 244 sentences (which contain 536 positive instances and 287 negative instances). See Table 3.

#### Unlabelled data

After choosing the training and testing sentences from the initial set of sentences, the remaining sentences have been used as unlabelled data. The unlabelled set contains 3440 sentences. A subset of these (408 sentences containing 823 instances which approximates the number found in the original training set) are used in the self-training step^4^.

### Training a model with the machine learning method

Now that we have a labelled training set, it is possible to train a model using a supervised machine learning method to be evaluated on the test set. We have applied the maximum entropy classifier developed for relationship identification (described above) [48] for our genotype-phenotype relationship identification application. A genotype-phenotype pair is represented by a set of features derived from a sentence. Tables 1 and 2 provide the list of features.

**Table 1:** List of dependency features

Features	Description
Relationship term	Root of the portion of the dependency tree connecting phenotype and genotype
Stemmed relationship term	Stemmed by Mallet
Relative position of relationship term	Whether it is before the first entity, after the second entity or between them
The relationship term combined with the dependency relationship	To consider the grammatical role of the relationship term in the dependency path.
The relationship term and its relative position	
Key term	Described in Ibn Faiz’s four step method [48]
Key term and its relative position	
Collapsed version of the dependency path	All occurrences of nsubj/nsubjpass are replaced with subj, rcmod/partmod with mod, prep x with x and everything else with O, a placeholder to indicate that a dependency has been ignored.
Second version of the collapsed dependency path	Only the prep_* of dependency relationships are kept.
Negative dependency relationship	A binary feature that shows whether there is any node in the path between the entities which dominates a *neg* dependency relationship. This feature is used to catch the negative relationships.
prep_between	A binary feature that checks for the existence of two consecutive prep_between links in a dependency path.

**Table 2:** List of syntactic and surface features

Features	Description
Syntactic features	
Stemmed version of relationship term in the Least Common Ancestor (LCA) node of the two entities	If the head^6^ of the LCA node of the two entities in the syntax tree is a relationship term then this feature takes a stemmed version of the head word as its value, otherwise it takes a NULL value.
The label of each of the constituents in the path between the LCA and each entity combined with its distance from the LCA node	
Surface features	
Relationship terms and their relative positions	The relationship terms between two entities or within a short distance (4 tokens) from them.

**Fig. 2:**
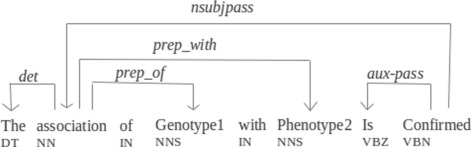
Dependency tree related to the sentence *“The association* *of Genotype1 with Phenotype2 is confirmed”*

Dependency parse trees can contain important information in the dependency path between two named entities. Figure 2 shows the dependency tree produced by the Stanford dependency parser^5^ for the sentence *“The association of Genotype1 with Phenotype2 is confirmed.”*. The dependency path between the phenotype and the genotype is “Genotype1-*prep_of*-association-*prep_with*-Phenotype2”. *Association* is the relationship term in this path and *prep_of* and *prep_with* are the dependency relationships related to it. The presence of a relationship term can be a signal for the existence of a relationship and its grammatical role along with its relative position gives valuable information about the entities involved in the relationship. Sometimes two entities are surrounded by more than one relationship term. *Key term* is introduced to find the relationship term which best describes the interaction. Ibn Faiz [48] used the following steps to find the key term: when one step fails the process continues to the next step, but if the key term is found in one step the following steps are ignored.

1. Any relationship term that occurs between the entities and dominates them both in the dependency representation is considered to be the key term.
2. A word is found that appears between the entities, dominates the two entities, and has a child which is a relationship term. That child is considered to be the key term.
3. Any relationship term that occurs on the left of the first entity or on the right of the second entity and dominates them both in the dependency representation is considered to be the key term.
4. A word appears on the left of the first entity or on the right of the second entity, dominates the two entities, and has a child which is a relationship term. That child is considered to be the key term.

#### Self-training algorithm

**Fig. 3:**
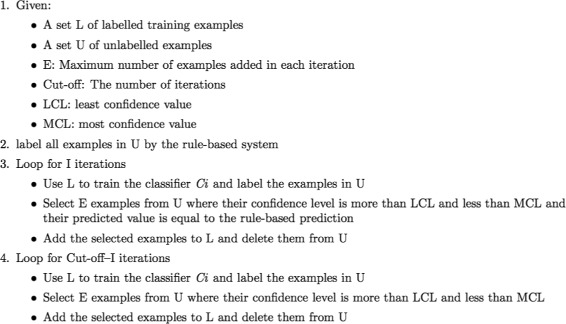
The self training process

The first model is trained using the training set and the machine learning method described earlier. To improve the performance of our model, a self-training process has been applied. Figure 3 outlines this process. This process starts with the provided labelled data and unlabelled data. The labelled data is used to train a model which is used to tag the unlabelled data. In most self-training algorithms the instances with the highest confidence level are selected to be added to the labelled data. However, as has been observed in some self-training algorithms, choosing the most confident unlabelled instances and adding them to the labelled data can cause overfitting [60]. We encountered a similar overfitting when we added the most confident unlabelled instances. So we considered the following two measures to select the best unlabelled instances.

- The confidence level must be in an interval. It must be more than a threshold *α* and less than a specified value *β*.
- The predicted value of the selected instances must be the same as their predicted value by the rule-based system.

In each iteration an at most upper-bounded number of instances are selected and added to the labelled data to prevent adding lots of incorrectly labelled data to the training set in the first iterations when the model is not powerful enough to make good predictions.

We used relationship identification output from the PPI-tailored rule-based tool as an added level of conservatism in the decision to add an unlabelled instance to the training set. It has only moderate performance on genotype-phenotype relationship identification. So, using this tool’s advice along with the confidence level means that the relationship must be of a more general nature than just genotype-phenotype relationships. However, at some point this conservatism holds the system back from learning broader types of relationships in the genotype-phenotype category. Therefore this selection factor is used only for the first *i* iterations, and after *i* iterations the best unlabelled data is chosen based only on the confidence level. Again, here, the confidence level must be in an interval.

This proposed self-training algorithm has been tried with various configurations and each variable in this process has been given several values. Each resulting model has been tried separately with our test set and the best system is selected based on its performance on the test set. In our best configuration 15 unlabelled instances are added to the labelled data in each iteration, in the first 5 iterations predictions made by the rule-based system are taken into account, the least confidence level is 85%, the highest confidence level is 92% and the process stops after 6 iterations.

## Results and discussion

The proposed machine-learned model has been evaluated using the separate test set manually annotated by a biochemistry graduate student. The distribution of our data (number of sentences and number of genotype-phenotype pairs in each set) is illustrated in Table 3. The numbers of positive instances and negative instances in the unlabelled data are not available.

**Table 3:** Distribution of data in our different sets

Data set	Sentences	Instances	Positive instances	Negative instances
Training set	509	845	576	269
Test set	244	823	536	287
Unlabelled data	408	823	N/A	N/A

Table 4 shows the results obtained by the supervised learning algorithm and the proposed self-training algorithm. The results of testing Ibn Faiz’s rule-based and machine learning-based relationship identification tools [48] originally configured to find protein-protein interactions have been included in the table for comparison purposes. Although these tools were not configured to be used for our application, as can be seen in the table, the PPI-configured tools, especially the rule-based system, have good precisions. This performance by the rule-based system led us to consider the rule-based predictions as one factor in choosing which unlabelled data to add to the labelled data. The recalls of the PPI-configured tools are quite low as one would expect. The precision results mean that there are some linguistic structures that are common between protein-protein and genotype-phenotype relationships and these structures are useful for distinguishing correct from incorrect relationship candidates.The low recall values indicate there are some genotype-phenotype relationship contexts which are specific to this type of relationship and the relation terms used to configure the general purpose relationship tools are key to finding these relationships.

**Table 4:** Evaluation results

Method	Precision	Recall	F-measure
Supervised learning method	76.47	77.61	77.03
Self-training method	77.70	77.84	77.77
PPI-configured ML-based tool	75.19	53.17	62.29
PPI-configured rule-based tool	77.77	38.04	51.09

As illustrated in Table 4, we get good performance by using a small initial training set and then we are able to gain a modest improvement by using our proposed self-training algorithm. The initial results with the small training set were: precision: 76.47, recall: 77.61, F-measure: 77.03. The self-training algorithm gave the following results: precision: 77.70, recall: 77.84, F-measure: 77.77. The self-training step provided only slightly more than 10% extra training examples (90 relationship instances added to the original 845 instances), so the modest performance improvement is not unexpected.

The following details will help to better appreciate these results. First, we have not attempted to find the best parameter settings by using the test set to determine these settings (this would lead to over-fitting to the test set). Rather, we have experimented with various parameter settings to understand how the semi-supervised method may work. We are using the modified learned model on the test set only to give precision and recall values to gauge the appropriateness of this technique. Second, instead of having a separate validation set and choosing the best model based on its performance with this set, every learned model (682 models were developed using 22 parameter settings and 1 to 31 iterations of the semi-supervised training step) has been tested with the test set. So, the results can be interpreted as: if a particular parameter setting and number of iterations of the semi-supervised algorithm would have produced the best model based on its performance on the validation set, this parameter setting and number of iterations of the semi-supervised algorithm would give the results based on its performance on the test set. Rather than reporting the best F-measure over all parameter settings, the data was studied to see certain trends. In particular, the reported values are for the best performing model in the semi-supervised iteration that happens before a decline in precision that is witnessed in almost all of the parameter settings. This we determined to be the sixth iteration. We chose this trend because the semi-supervised method at this point had provided the best ratio of true to false positives which we considered a worthwhile goal. Although some parameter settings performed better in terms of precision than these reported results, it was felt that using this (almost) global trend in precision as a cutoff point would be a better mark of the performance rather than looking solely at a single parameter setting that might be seen to be over-fitted to the test set.

**Fig. 4:**
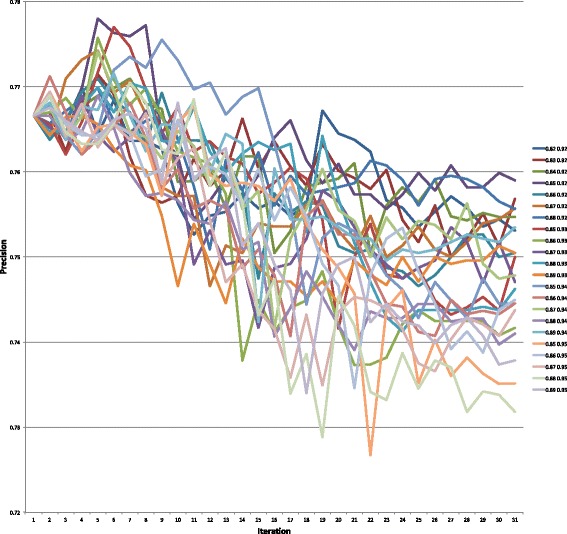
Precision values on the test set for all 22 parameter settings for 31 semi-supervised learning iterations

**Fig. 5:**
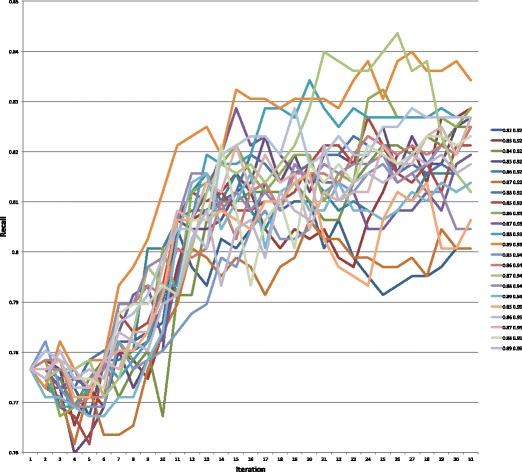
Recall values on the test set for all 22 parameter settings for 31 semi-supervised learning iterations

**Fig. 6:**
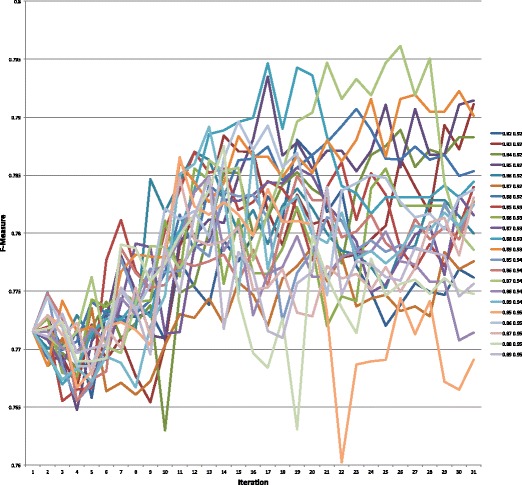
F-measure values on the test set for all 22 parameter settings for 31 semi-supervised learning iterations

**Fig. 7:**
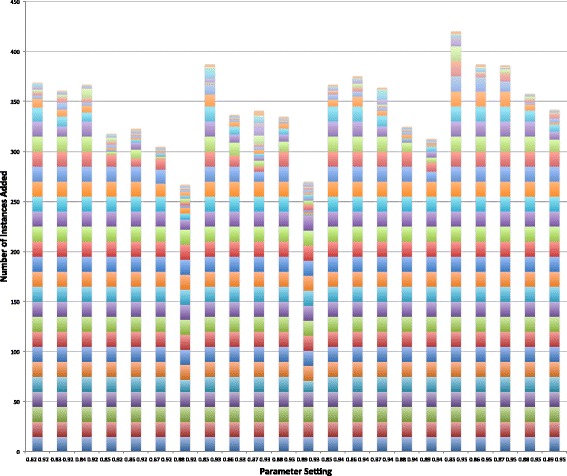
Instances added for all 22 parameter settings for 31 semi-supervised learning iterations on the test set

Graphs of the precision, recall, and F-measure values for each parameter setting for the 31 iterations of the semi-supervised learning algorithm are presented in Figs. 4, 5, and 6, respectively. Table 5 highlights the maximum values for each of these measures. The values for each of these measures for all 682 parameter settings can be found in https://github.com/mkhordad/Pheno-Geno-Extraction. There are two general trends in all of the parameter settings that we tried. First, there is a short increase in precision followed by a slow decline in this measure. Second, a short decline in recall is followed by a general increase in this measure until the point (approximately iteration 15 to 17) when few new instances are being added to the training set. See Fig. 7 for a presentation of the addition of instances to the training set for each parameter setting. It should be noted that shortly after iteration 15, few instances are available to be added to the training set. The minimum and maximum value range proves to be too narrow in some instances, but eventually all experimental settings lack instances to add. The precision and recall curves tend to flatten out at about this point. It would be interesting to see how an increase in unlabelled instances would affect the outcome of the semi-supervised learning.

**Table 5:** Maximum values for precision, recall, and F-measure

	Precision		Recall		F-Measure	
Parameter setting	Maximum value	Iteration	Maximum value	Iteration	Maximumvalue	Iteration
0.82 0.92	0.7699	4	0.8138	17	0.7880	19
0.83 0.92	0.7714	5	0.8287	31	0.7911	31
0.84 0.92	0.7709	7	0.8250	30	0.7889	26
0.85 0.92	0.7780	5	0.8268	31	0.7935	17
0.86 0.92	0.7709	5	0.8156	13	0.7870	12
0.87 0.92	0.7743	5	0.8063	20	0.7788	20
0.88 0.92	0.7698	5	0.8231	23	0.7907	23
0.85 0.93	0.7770	6	0.8268	24	0.7870	12
0.86 0.93	0.7757	5	0.8324	25	0.7856	25
0.87 0.93	0.7689	4	0.8287	15	0.7857	19
0.88 0.93	0.7704	7	0.8343	20	0.7946	17
0.89 0.93	0.7665	1	0.8399	27	0.7923	30
0.85 0.94	0.7755	9	0.8250	31	0.7836	14
0.86 0.94	0.7712	2	0.8250	31	0.7849	19
0.87 0.94	0.7741	5	0.8436	26	0.7961	26
0.88 0.94	0.7689	5	0.8194	25	0.7849	13
0.89 0.94	0.7715	6	0.8156	13	0.7892	13
0.85 0.95	0.7694	2	0.8156	20	0.7866	11
0.86 0.95	0.7688	2	0.8287	19	0.7896	15
0.87 0.95	0.7694	2	0.8268	31	0.7848	11
0.88 0.95	0.7705	7	0.8231	28	0.7875	14
0.89 0.95	0.7681	10	0.8212	21	0.7848	13

Recalling the work of Singhal et al. [41], they investigated disease-gene-variant triplets, which is close to the focus of this paper, and they provided precision, recall, and F-measure values based on the performance of their system on two datasets curated from human-annotated PubMed articles concerning prostate and breast cancer. The precision, recall, and F-measure results were 0.82, 0.77, and 0.794, and 0.742, 0.73, and 0.74, respectively for the two datasets. Also recalling the work of Bokharaeian et al. [47], they investigated relationships between SNPs and phenotypes. Looking at their reported results that are closest to what is reported here, they achieve precision up to 69.2, recall up to 68.7, and F-measure up to 71.3. With the understanding that the datasets are different and the relationships being identified are closely related but not exactly the same, we can say that the method presented here, which is based only on the natural language text surrounding the genotype-phenotype relationship, compares favourably with the results obtained by these other methods.

Looking forward, some improvements to the current model can be suggested. Some of these improvements are typical of the machine-learning paradigm. First is the balance of positive and negative examples in the training set. While we tried to add some negative sentences to our data to make it more balanced, Table 3 shows that our data is still biased: the number of negative instances is less than the number of positive instances. A more balanced training set is likely to improve the performance of the trained model. Second, the quality of the original set of examples which forms the seed for the self-training algorithm affects the ability of that algorithm to increase the size of our training set. Because the best results were reached only after 6 iterations, the last training set has only 935 instances. Our suggestion is to add more manually annotated sentences to the original seed training set, so that the first model made by this set makes better predictions with a stronger level of confidence.

In addition to these methodological improvements, the similarity of false positives and false negatives can indicate some aspects of the problem to focus on. For instance, our system incorrectly finds relationships in sentences which address the main objective of the research being discussed, i.e., those sentences suggesting the possibility of a relationship rather than stating a relationship. Finding and ignoring such sentences would improve the results.

As mentioned before, certain relationships contained in the *Phenominer* corpus are undetectable in the test set data because the relationship identification system does not have the appropriate biological and linguistic knowledge to recognize them. Table 6 shows the results after deleting the *Phenominer* sentences from our test set. The improved results (precision: 80.05, recall: 81.07, F-measure: 80.55) demonstrate the true performance of the relationship tool to identify relationships for which it was constructed to find. Detecting these problematic relationships would require some significant changes to the system.

**Table 6:** Results after deleting *Phenominer* sentences from the test set

Method	Precision	Recall	F-measure
Supervised learning method	80.20	79.79	80.00
Self-training method	80.05	81.07	80.55

First, the current system does not recognize relationships that deal with sub-cellular phenotypes. To include this type of phenotype, biomedical knowledge will need to be enhanced to identify these phenotypes in the text. Our system was built to consider only clinically observable phenotypes. Additionally, the linguistic knowledge will need to be supplemented because the direction of this relationship is different. Second, the current system is not able to extract complicated relations where a pronoun refers to a phenotype or a genotype in the same sentence or the previous sentences (anaphora), or where a non-explicit noun phrase is used to refer (e.g., *the gene*), or where a part of or the whole genotype or phenotype is omitted (ellipsis) in a sentence. For example in the following sentence *“Serum levels of anti-gp70 Abs-7 were closely correlated with the presence of renal disease-16, more so than anti-dsDNA Abs-24.”* only the relationship between *anti-gp70 Abs-7* and *renal disease-16* is identified by our system but the more complicated relationship between *renal disease-16* and *anti-dsDNA Abs-24* is missed. Resolving these problems will require a more sophisticated linguistic model, the focus of computational linguistics research generally.

## Conclusions

To summarize, our contributions in this paper are the following:

- Reconfiguring a generic relationship identification method to perform genotype-phenotype relationship identification.
- Proposing a semi-automatic method for making a small training set using two relationship identification tools.
- Developing a self-training algorithm to enlarge the training set and improve the genotype-phenotype relationship identification results.
- Analysing the results and specifying the types of sentences and relationships that our system has poor performance finding and giving some suggestions on how to improve the results.

In conclusion, we have generated a machine-learned model dedicated solely to the identification of genotype-phenotype relationships mentioned in biomedical text using only the surrounding text. With a test corpus, we have provided a baseline measure of precision, recall, and F-measure for future comparison. An analysis of the false negatives and false positives from this corpus have suggested some natural language processing enhancements that would decrease the false negative and false positive rates. From a biological perspective, determining the type of relationship, e.g., does the relationship describe a direct expression of a gene or is the relationship indicative of a pathway effect, would be an important aspect of the relationship to mine from the text and is an interesting next research direction to consider.

## Endnotes

^1^ A directed graph representing dependencies of words in a sentence.

^2^ Seven verbs from [9] are not found in [48]. The approximately 270 relationship words (808 surface forms) can be found in https://github.com/mkhordad/Pheno-Geno-Extraction. These words have a good overlap with the current relations in the UMLS Semantic Network that were used in Sharma et al.’s verb-centric approach [61].

^3^ Genotype-phenotype pairs that have a relationship are the positive instances. Genotype-phenotype pairs that do not have a relationship are the negative instances. The sentences mentioned have both positive and negative instances.

^4^ Each self-training iteration requires each sentence to be evaluated using the current model. Using the full unlabelled set proved to be too computationally expensive for the experimental setting, so a subset was used instead.

^5^ http://nlp.stanford.edu/software/stanford-dependencies.shtml

^6^ Collins’ head finding rule [62] has been used.

## References

[1] McKusick V (2007). Mendelian Inheritance in Man and its online version, OMIM. Am J Hum Genet.

[2] Sekimizu T, Park HS, Tsujii J (1998). Identifying the interaction between genes and gene products based on frequently seen verbs in MEDLINE abstracts. Genome Inform.

[3] Temkin JM, Gilder MR (2003). Extraction of protein interaction information from unstructured text using a context-free grammar. Bioinformatics (Oxford, England).

[4] Coulet A, Shah NH, Garten Y, Musen MA, Altman RB (2010). Using text to build semantic networks for pharmacogenomics. J Biomed Inform.

[5] Ng S, Wong M (1999). Toward routine automatic pathway discovery from on-line scientific text abstracts. Genome Inform.

[6] Huang M, Zhu X, Hao Y, Payan DG, Qu K, Li M (2004). Discovering patterns to extract protein–protein interactions from full texts. Bioinformatics.

[7] Craven M (1999). Learning to extract relations from MEDLINE. AAAI-99 Workshop on Machine Learning for Information Extraction.

[8] Katrenko S, Adriaans P (2007). Learning relations from biomedical corpora using dependency trees. Knowledge Discovery and Emergent Complexity in Bioinformatics, First International Workshop (KDECB 2006), Volume 4366 of Lecture Notes in Computer Science.

[9] Rindflesch TC, Libbus B, Hristovski D, Aronson AR, Kilicoglu H (2003). Semantic relations asserting the etiology of genetic diseases. AMIA Annual Symposium Proceedings.

[10] Fundel K, Küffner R, Zimmer R (2007). RelEx - Relation extraction using dependency parse trees. Bioinformatics.

[11] Bui QC, Katrenko S, Sloot PMA (2011). A hybrid approach to extract protein-protein interactions. Bioinformatics.

[12] Leroy G, Chen H, Martinez JD (2003). A shallow parser based on closed-class words to capture relations in biomedical text. J Biomed Inform.

[13] Klein TE, Chang JT, Cho MK, Easton KL, Fergerson R, Hewett M, Lin Z, Liu Y, Liu S, Oliver DE, Rubin DL, Shafa F, Stuart JM, Altman RB (2001). Integrating genotype and phenotype information: an overview of the PharmGKB project. Pharmacogenomics J.

[14] de Marnee MC, Manning CD. Stanford typed dependencies manual. 2015. (Accessed 1 May 2015) [http://nlp.stanford.edu/software/dependencies_manual.pdf].

[15] Yakushiji A, Tateisi Y, Miyao Y, Tsujii J (2001). Event extraction from biomedical papers using a full parser. Pacific Symposium on Biocomputing.

[16] Cormen TH, Leiserson CE, Rivest RL, Stein C (2001). Introduction to Algorithms, Second Edition.

[17] Marcotte EM, Xenarios I, Eisenberg D (2001). Mining literature for protein-protein interactions. Bioinformatics.

[18] Stephens M, Palakal M, Mukhopadhyay S, Raje R, Mostafa J (2001). Detecting gene relations from MEDLINE abstracts. Pacific Symposium on Biocomputing.

[19] Benoit G, Stapley BJ (2000). Biobibliometrics: Information retrieval and visualization from co-occurrences of gene names in Medline abstracts. Pacific Symposium on Biocomputing.

[20] Jenssen T, Laegreid A, Komorowski J, Hovig E (2001). A literature network of human genes for high-throughput analysis of gene expression. Nat Genet.

[21] Rosario B, Hearst MA (2004). Classifying semantic relations in bioscience texts. Proceedings of the 42nd Annual Meeting of the Association for Computational Linguistics.

[22] Frunza O, Inkpen D (2010). Extraction of disease-treatment semantic relations from biomedical sentences. Proceedings of the 2010 Workshop on Biomedical Natural Language Processing.

[23] Frunza O, Inkpen D, Tran T (2011). A machine learning approach for identifying disease-treatment relations in short texts. IEEE Trans Knowl Data Eng.

[24] Abacha AB, Zweigenbaum P (2011). Automatic extraction of semantic relations between medical entities: a rule based approach. J Biomed Semant.

[25] Abacha AB, Zweigenbaum P (2011). A hybrid approach for the extraction of semantic relations from MEDLINE abstracts. Proceedings of the 12th International Computational Linguistics and Intelligent Text Processing Conference Part II, CICLing 2011, Volume 6609 of Lecture Notes in Computer Science.

[26] Yang H, Swaminathan R, Sharma A, Ketkar V, D’Silva J (2011). Mining biomedical text towards building a quantitative food-disease-gene network. Learning Structure and Schemas from Documents, Volume 375 of Studies in Computational Intelligence.

[27] Banko M, Cafarella MJ, Soderland S, Broadhead M, Etzioni O (2007). Open information extraction from the Web. Proceedings of the 20th International Joint Conference on Artifical Intelligence, IJCAI’07.

[28] Fader A, Soderland S, Etzioni O (2011). Identifying relations for open information extraction. Proceedings of the Conference on Empirical Methods in Natural Language Processing, EMNLP ’11.

[29] Mausam MS, Bart R, Soderland S, Etzioni O (2012). Open language learning for information extraction. Proceedings of the 2012 Joint Conference on Empirical Methods in Natural Language Processing and Computational Natural Language Learning, EMNLP-CoNLL ’12.

[30] Nguyen N, Miwa M, Tsuruoka Y, Chikayama T, Tojo S (2015). Wide-coverage relation extraction from MEDLINE using deep syntax. BMC Bioinformatics.

[31] Xu Y, Kim MY, Quinn K, Barbosa D, Goebel R (2013). Open information extraction with tree kernels. Proceedings of the 2013 Conference of the North American Chapter of the Association for Computational Linguistics: Human Language Technologies.

[32] de Sá Mesquita F, Schmidek J, Barbosa D (2013). Effectiveness and efficiency of open relation extraction. Proceedings of the 2013 Conference on Empirical Methods in Natural Language Processing, EMNLP 2013.

[33] Lamblin P, Bengio Y. Important gains from supervised fine-tuning of deep architectures on large labeled sets. In: NIPS’2010 Deep Learning and Unsupervised Feature Learning Workshop: 2010. (https://deeplearningworkshopnips2010.wrdpress.com/schedule/acceptedpapers), WordPress.com.

[34] Krizhevsky A, Sutskever I, Hinton G (2012). Imagenet classification with deep convolutional neural networks. Advances in Neural Information Processing Systems.

[35] Socher R, Lin CC, Ng A, Manning C (2011). Parsing natural scenes and natural language with recursive neural networks. Proceedings of the 28th International Conference on Machine Learning, ICML 2011.

[36] Li C, Song R, Liakata M, Vlachos A, Seneff S, Zhang X (2015). Using word embedding for bio-event extraction. Proceedings of the 2015 Workshop on Biomedical Natural Language Processing.

[37] Miwa M, Bansal M (2016). End-to-end relation extraction using LSTMs on sequences and tree structures. Proceedings of the 54th Meeting of the Association for Computational Linguistics.

[38] Jiang Z, Jin L, Li L, Qin M, Qu C, Zheng J, Huang D. A CRD-WEL System for Chemical-disease Relations Extraction. In: Proceedings of the Fifth BioCreative Challenge Evaluation Workshop: 2015. p. 317–326. www.biocreative.org.

[39] Liu F, Chen J, Jagannatha A, Yu H (2016). Learning for biomedical information extraction: Methodological review of recent advances. CoRR.

[40] Doughty E, Kertesz-Farkas A, Bodenreider O, Thompson G, Adadey A, Peterson T, Kann MG (2011). Toward an automatic method for extracting cancer- and other disease-related point mutations from the biomedical literature. Bioinformatics.

[41] Singhal A, Simmons M, Lu Z (2016). Text mining genotype-phenotype relationships from biomedical literature for database curation and precision medicine. PLoS Comput Biol.

[42] Verspoor KM, Heo GE, Kang KY, Song M (2016). Establishing a baseline for literature mining human genetic variants and their relationships to disease cohorts. BMC Med Inform Decis Making.

[43] Mahmood AA, Wu T, Mazumder R, Vijay-Shanker K (2016). DiMeX: A text mining system for mutation-disease association extraction. PLoS ONE.

[44] Opap K, Mulder N (2017). Recent advances in predicting gene–disease associations. F1000Research.

[45] Korbel JO, Doerks T, Jensen LJ, Perez-Iratxeta C, Kaczanowski S, Hooper SD, Andrade MA, Bork P (2005). Systematic association of genes to phenotypes by genome and literature mining. PLoS Biol.

[46] Goh CS, Gianoulis TA, Liu Y, Li J, Paccanaro A, Lussier YA, Gerstein M (2006). Integration of curated databases to identify genotype-phenotype associations. BMC Genomics.

[47] Bokharaeian B, Diaz A, Taghizadeh N, Chitsaz H, Chavoshinejad R (2017). SNPPhenA: A corpus for extracting ranked associations of single-nucleotide polymorphisms and phenotypes from literature. J Biomed Semant.

[48] Ibn Faiz MS (2012). Discovering higher order relations from biomedical text. Master’s thesis.

[49] Riloff E, Shepherd J (1997). A corpus-based approach for building semantic lexicons. Proceedings of the 2nd Conference on Empirical Methods in Natural Language Processing.

[50] Riloff E, Jones R (1999). Learning dictionaries for information extraction by multi-level bootstrapping. Proceedings of the 16th National Conference on Artificial Intelligence and the 11th Innovative Applications of Artificial Intelligence Conference.

[51] Carlson A, Betteridge J, Hruschka Jr E, Mitchell T (2009). Coupling semi-supervised learning of categories and relations. Proceedings of the NAACL HLT Workshop on Semi-supervised Learning for Natural Language Processing.

[52] Ng V, Cardie C (2003). Weakly supervised natural language learning without redundant views. Proceedings of the 2003 Conference of the North American Chapter of the Association for Computational Linguistics on Human Language Technology - Volume 1, NAACL ’03.

[53] Curran JR, Murphy T, Scholz B (2007). Minimising semantic drift with mutual exclusion bootstrapping. Proceedings of the 10th Meeting of the Pacific Association for Computational Linguistics, PACLING 2007.

[54] McIntosh T, Curran JR (2008). Weighted mutual exclusion bootstrapping for domain independent lexicon and template acquisition. Proceedings of the Australasian Language Technology Association Workshop.

[55] Clark S, Curran JR, Osborne M (2003). Bootstrapping POS taggers using unlabelled data. Proceedings of the Seventh Conference on Natural Language Learning at HLT-NAACL 2003 - Volume 4, CONLL ’03.

[56] Khordad M, Mercer RE, Rogan P (2012). A machine learning approach for phenotype name recognition. Proceedings of the 24th International Conference on Computational Linguistics, COLING 2012.

[57] Collier N, Tran MV, Le HQ, Oellrich A, Kawazoe A, Hall-May M, Rebholz-Schuhmann D (2012). A hybrid approach to finding phenotype candidates in genetic texts. Proceedings of the 24th International Conference on Computational Linguistics, COLING 2012.

[58] Ohta T, Tateisi Y, Kim JD (2002). The GENIA corpus: An annotated research abstract corpus in molecular biology domain. Proceedings of the Human Language Technology Conference.

[59] Leaman R, Gonzalez G (2008). BANNER: An executable survey of advances in biomedical named entity recognition. Pacific Symposium on Biocomputing.

[60] Zhu X, Goldberg AB, Brachman R, Dietterich T (2009). Introduction to Semi-Supervised Learning. Synthesis Lectures on Artificial Intelligence and Machine Learning.

[61] Sharma A, Swaminathan R, Yang H (2010). A Verb-centric Approach for Relationship Extraction in Biomedical Text. Proceedings of the 2010 IEEE Fourth International Conference on Semantic Computing.

[62] Collins M (2003). Head-driven statistical models for natural language parsing. Comput Linguist.

